# FHIR-AgentEval: A Modular Sandbox for Benchmarking Clinical LLM Agents with an Evaluation of Memory-Augmented Configurations

**DOI:** 10.21203/rs.3.rs-8746188/v1

**Published:** 2026-02-09

**Authors:** Youssef Mokssit, Kamalakkannan Ravi, Mengshu Nie, Junyoung Kim, Cong Liu

**Affiliations:** Boston Children’s Hospital; Boston Children’s Hospital; Boston Children’s Hospital; Boston Children’s Hospital; Boston Children’s Hospital

## Abstract

Healthcare data exchange increasingly relies on HL7 FHIR, but FHIR’s implementation complexity creates barriers for clinical workflows. Large language model (LLM) agents could bridge this gap by translating natural language requests into structured FHIR operations, yet their reliability remains unproven. We present FHIR-AgentEval, an extensible evaluation sandbox comprising 43 modular tasks for benchmarking LLM agents on realistic appointment management and genetic testing workflows. Each task executes against a resettable FHIR server with custom deterministic validation of both agent responses and resulting server state. We run an ablation study of five agent configurations, varying access to an on-demand FHIR R4 specifications server and long-term memory trained with or without specification grounding. Across four experimental settings, memory consistently improves task success and reduces strategic failures such as incorrect tool selection and resource-type confusion. On held-out tasks, the best memory configuration improves success by 9.1% over baseline, offering a potential pathway toward more robust clinical deployment.

## Introduction

Healthcare data exchange is increasingly standardized on HL7 FHIR (Fast Healthcare Interoperability Resources), driven by regulatory mandates and broad adoption by major Electronic Health Record (EHR) vendors^1–3^. Clinicians now interact with diverse health IT systems (EHR interfaces, appointment system, lab ordering portals, etc.), and under the hood these workflows rely on FHIR API calls encoding clinical actions (e.g. retrieving patient data or placing an order). Despite FHIR’s promise of seamless interoperability, its implementation complexity poses a well-documented barrier for developers and healthcare IT teams^4,5^. Each FHIR resource comes with numerous fields, interdependencies, and strict schemas, resulting in a steep learning curve and lengthy development cycles even for experienced engineers. As a result, many clinical workflows today still rely on rigid, pre-programmed integration logic or vendor-provided hard-coded modules, rather than dynamic, on-demand requests. There is a pressing need for solutions that lower the barrier to harnessing FHIR’s full flexibility, allowing healthcare practitioners and systems to execute custom requests without deep technical overhead^6^.

Recent advances in large language models (LLMs) offer a promising avenue to bridge this usability gap. LLM-based “agents” can interpret free-text requests and translate them into precise API calls, essentially serving as a natural language interface on top of FHIR infrastructure. Early demonstrations in the digital health domain underscore this potential. For example, LLMonFHIR uses LLMs to let patients query and understand their health records in natural language, across any level of complexity or even in different languages^7^. On a pilot evaluation, physicians rated the accuracy and clarity of LLMonFHIR’s answers to patient-specific queries very highly (median 5 out of 5), highlighting how an LLM can successfully retrieve and summarize FHIR-based data^7^. Likewise, researchers have explored using LLMs to convert unstructured clinical text into structured FHIR resources (FHIR-GPT) as a means to enhance interoperability^8^. On a broader scale, large tech initiatives such as Google’s Personal Health Agent are experimenting with conversational agents that act on medical records data to answer real clinical questions^9^. These efforts all point to an emerging paradigm: LLM-driven agents have the potential to “understand” a user’s intent and perform the corresponding clinical data “action”, from retrieving a patient’s lab results to writing a new order, without the user needing to craft a complex FHIR query manually.

Despite this promise, reliability and safety remain critical challenges. Unlike static queries, an autonomous agent navigating live clinical data carries the risk of mistakes (e.g. retrieving incorrect records or ordering a wrong medication), which in healthcare can have high-stakes consequences. Therefore, benchmarks are needed to evaluate how a FHIR agent performs before it can be deployed. Recent benchmarking studies reveal that state-of-the-art LLM agents are still far from infallible in complex medical environments.

MedAgentBench^10^, for instance, evaluated several cutting-edge models on 300 realistic EHR tasks. The best model achieved only about 70% success in executing the tasks correctly, with performance dropping significantly in scenarios requiring nuanced reasoning or multi-step workflows (sequences of actions) rather than simple question answering^10^. This underscores that current LLM agents often falter on complex, multi-step clinical workflows and that substantial improvement is needed before such agents can be trusted in real patient care. In short, while LLMs can say the right things in medical Q&A, enabling them to “do” the right things (i.e. take correct actions in an EHR) sets a much higher bar. The community has recognized the need for rigorous benchmarks with sandbox capabilities that allow agents to execute safely, enabling the pre-deployment evaluation.

In this study, we introduce FHIR-AgentEval, a modular evaluation sandbox for benchmarking agent performance on realistic FHIR-based clinical workflow tasks. We evaluate five ReAct^11^-style agent configurations that vary access to auxiliary resources, including an ondemand FHIR R4 specifications server and a Reflexion^12^-inspired long-term memory server trained offline with or without specification grounding. By comparing these configurations under various task conditions, we aim to identify the features most improve accuracy and reliability in executing FHIR workflows.

## Methodology

### Sandbox overview

We use the following terminology throughout the paper. A *task* is a modular benchmark scenario defined by a prompt template with parameter placeholders, environment setup, and deterministic validation logic. A *task variation* is a concrete instance of a task created by instantiating these placeholders with specific values. The resulting *task description* is the fully specified prompt and represents the only context provided to the agent. An *agent (or task) run* refers to a complete execution of a single task variation by a given agent configuration, including environment setup, agent interaction, validation, and logging.

**Figure 1:**
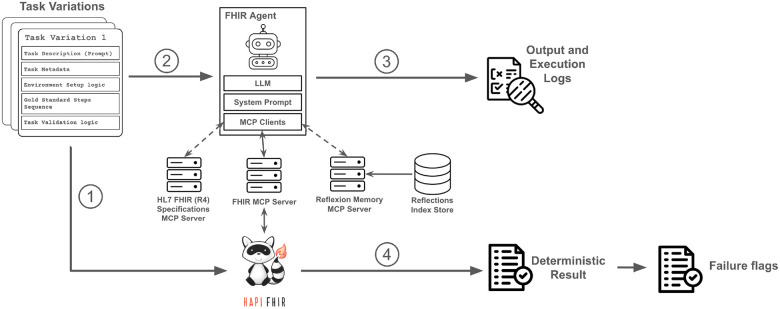
FHIR-Agent Eval Evaluation sandbox overview and task run process. Dashed arrows denote an optional configuration-specific connection, while solid arrows denote a mandatory connection. The main steps of a task run are: 1) Environment setup. 2) Prompting the agent with the task description. 3) Extracting the agent output and execution logs. 4) Validating task success against the HAPI FHIR server state and generating a run report.

Our evaluation sandbox (Figure 1) combines this curated task set with an end-to-end execution and validation pipeline. Any Model Context Protocol (MCP)^13^-compatible agent can be plugged into the sandbox and evaluated under identical conditions. Each run produces detailed execution logs and contributes to an aggregate evaluation report.

### Task structure.

Our benchmark includes 43 tasks based on appointment management and genetic testing workflows. Each task represents a realistic clinical scenario and is designed for reuse by injecting different parameters (e.g., patient, provider, date) at runtime to generate *task variations*.

**Figure 2:**
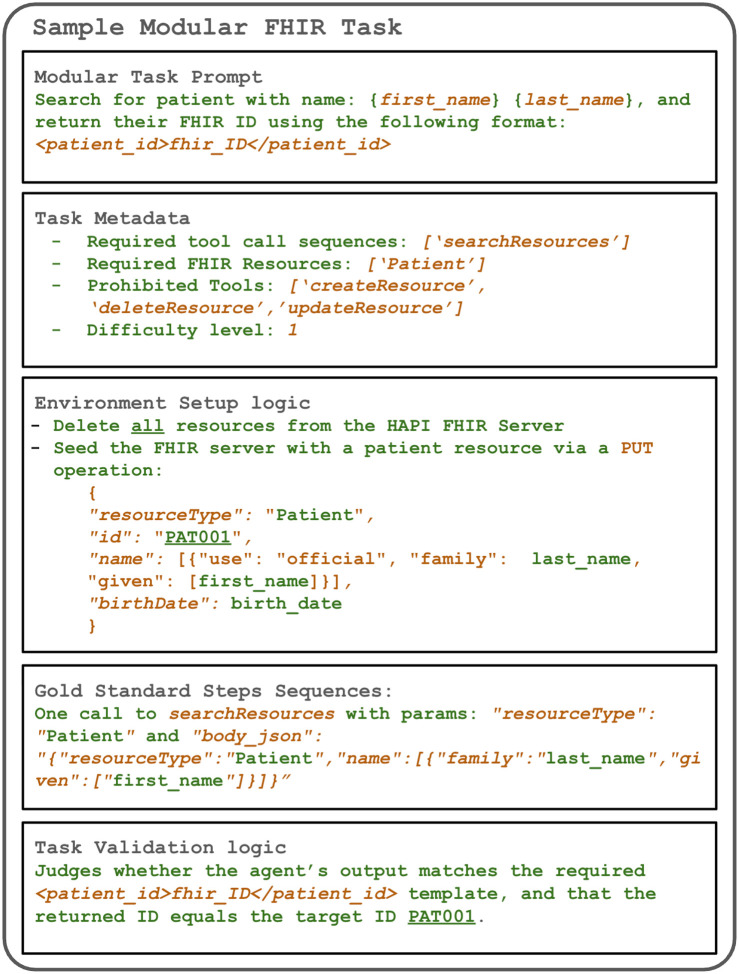
Main components of a modular task in our benchmark.

Figure 2 illustrates the components required for a fully specified task.

- *Prompt template:* a free-text prompt with placeholders for injectable details. This prompt constitutes the entire context that an agent receives at runtime. It includes the task’s core purpose, formatting requirements, and a current date/time block generated at runtime. Once instantiated, it becomes the *task description* given to the agent.
- *Task metadata:* Evaluation-specific constraints and success criteria, including required FHIR resources, expected or prohibited tool calls, and a manually assigned difficulty score from 1 to 3.
- *Environment setup:* Before each run, the HAPI FHIR^14^ server is reset and then seeded with task-specific prerequisite resources, along with additional irrelevant resources to emulate realistic EHR complexity.
- *Gold standard tool call sequences:* A reference implementation by human engineers that performs the optimal sequence of FHIR operations. This is used to verify task feasibility and, in some cases, as a reference during validation.
- *Task validator:* A deterministic validator first checks whether the agent’s output matches the required response format and extracts returned resource identifiers. Validation then depends on task type: query-only tasks compare returned IDs against expected targets, while tasks involving creation or modification (23 out of 43 tasks) additionally verify server-side resource state and field-level changes. For those 23 tasks, we also apply a relaxed “light validator” that verifies existence of the required fields only without enforcing exact values.

#### Task curation process.

**Figure 3:**
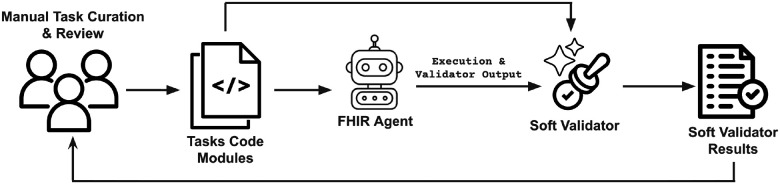
Task curation process. Soft validator feedback is used to iteratively identify and correct biases in each task’s deterministic validation logic.

We manually curated the benchmark by defining clinical administrative scenarios and implementing them as tasks (Figure 3). Each task includes a human-written deterministic validator based on the intended results. To account for variability in FHIR implementation, we use a “soft validator”, an LLM judge that reviews the task definition, execution logs, and the deterministic validation results. The soft validator assigns one of three outcome labels based on its assessment:

1. *agreement*: this means that the soft validator agrees with the task’s deterministic validator on the success or the failure of the task run.
2. *validator_too_strict*: this label means that the deterministic validator judges a task run as being a failure, however, the soft validator deems the validator overly strict (e.g. a field can be an optional).
3. *validator_too_loose*: means that the deterministic validator marks a run as a success, but the soft validator disagrees.

For each task, we run a single task variation using a LangChain OpenAI tools agent^15^ (GPT-4.1^16^), then use the soft validator to identify potential issues. We iteratively review soft validator feedback and inspect runs, and update the deterministic validators and prompt templates in some cases until their judgments align. The soft validator is used only during task curation. All experiments (see below) rely solely on the finalized deterministic validator.

### MCP servers

Our evaluation sandbox includes a set of MCP servers:

- *FHIR MCP server:* This server acts a gateway to our HAPI FHIR backend by exposing 5 tools: *createResource, searchResources, getResourceById, updateResource*, and *deleteResource*, which provide an interface over the HTTP supported by HAPI FHIR.
- *FHIR R4 specifications MCP server*: This server serves specifications sourced from the official HL7 FHIR R4 core package (hl7.fhir.r4.core^17^). It enables on-demand retrieval of resource types, structure definitions, and search parameters. It allows agents to dynamically inspect the structure and constraints of FHIR resources, ensuring that requests are constructed with the correct fields, data types, cardinality, and search syntax in accordance with FHIR R4 standards. In our setup, the FHIR R4 specifications MCP server exposes four tools*: listResources*, *getStructureDefinition*, *getSearchParams,* and *getDataTypeDefinition:*
- *Reflexion long-term memory MCP server:* This server provides tips and lessons learned during the Reflexion-inspired training (see Reflexion Training process). Agents can query it for either micro reflections; tips about a (FHIR resource, operation) pair, or for macro reflections which contain higher-level strategic planning for the task.

### Implementation of agent execution

We implement each configuration around a LangChain OpenAI tools agent (GPT 4.1-mini^18^) that follows a ReAct-style execution loop. Each run is capped at 15 iterations, where an iteration typically consists of a tool call followed by an observation. The run stops for one of three reasons: (1) the agent emits a final answer because it believes the task is complete, (2) the agent emits a final answer after encountering repeated errors or insufficient information, or (3) the agent reaches the maximum iterations limit.

To execute a task variation, we first instantiate it with specific values and prepare the HAPI FHIR server using task-specific setup. The task description (i.e. prompt) is then given to an agent, which interacts with the HAPI FHIR server via MCP layer and may access additional MCP servers depending on the configuration. After the agent finishes, we collect its outputs and logs and use the task’s deterministic validator to determine success.

### Reflexion training process

**Figure 4:**
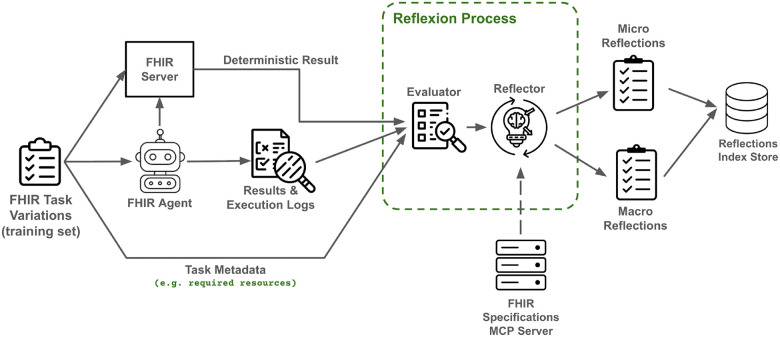
Reflexion-inspired training process. Agent runs on training task variations are evaluated and reflected upon to generate operation-specific micro reflections and task-level macro reflections for future retrieval.

We adopted a Memory Augmented Generation^19^ framework, where memory is a persistent store distilled during a separate training phase and retrieved at evaluation time across tasks and variations. [Figure 4] shows the process of our Reflexion-inspired training. It runs a simple ReAct-style agent (based on GPT-4.1) on a set of training tasks, using up to two training variations per task and allowing up to two trials per variation, with early stopping on success. After each run, execution logs, validation outcomes, and task metadata are sent to a Reflexion pipeline with two components. (1) An *evaluator* (GPT-4.1) critiques (**Supplementary File Prompt 1**) the run and provides structured feedback on execution quality, constraint adherence, and failure modes. (2) A *reflector (GPT*-o4-mini^20^) turns the feedback, execution traces, and deterministic validator outcomes into reusable lessons (**Supplementary File Prompt 2**). The reflector can optionally query the FHIR R4 specifications MCP server to ensure reflections adhere to the standard. This module generates two types of reflections:

- *Micro reflections:* capture operational best practices for specific (FHIR resource, operation) pairs. These reflections typically pertain to tool hygiene and FHIR R4 specifications (e.g. offering guidance on proper JSON structure to create or search for a specific FHIR resource).
- *Macro reflections:* offer higher-level strategic advice about the entire task run. These reflections focus on overall planning, tool sequencing, tool output filtering logic, and adherence to the task’s constraints. They are designed to help the agent follow more effective strategies when faced with similar task scenarios in the future.

All reflections are indexed using a dual FAISS^21^-based memory store, with *macro reflections* embedded and retrieved via semantic similarity, and *micro reflections* stored with structured metadata for exact (FHIR resource, operation) matching.

### Experiment setup

We evaluated five agent configurations (Table 1) under four training settings to study the impact of training and agent design choices. The four training settings are:

- *Experiment 1*: Train Reflexion on one variation per task and evaluate on the same variations.
- *Experiment 2*: Train on two variations per task and evaluate on three unseen variations per task.
- *Experiment 3*: Same as Experiment 2, but task descriptions are paraphrased using GPT-4.1 during both training and evaluation.
- *Experiment 4*: Train on two variations of 21 tasks and evaluate on three variations of the remaining 22 held-out tasks, with paraphrasing enabled throughout.

## Results

### Main results based on configuration.

**Figure 5:**
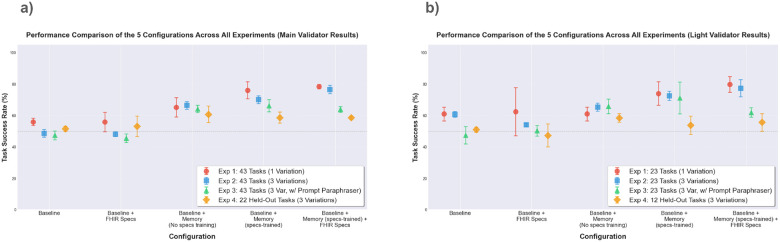
Task success rates across agent configurations and experimental settings. Figure 5ashows performance of five agent configurations on all 43 clinical workflow tasks under four experimental settings that vary training scope, task overlap, and number of task variations per task, with success determined by each task’s main deterministic validator. Figure 5b shows performance on the subset of 23 tasks involving FHIR resource creation or modification (e.g. appending a new field to a FHIR resource), evaluated using light validation that verifies required fields and structure without enforcing exact content values. Error bars represent standard deviation over three independent runs.

Figure 5a reports the mean task success rate (± standard deviation) over three runs. Access to the memory consistently improve performance across all experiments. Access to the FHIR R4 specifications alone does not significantly improves performance, but using the specs during Reflexion training often boosts results across experiments. In Experiment 1, which serves as a positive control, increased MCP access improves performance, with memory providing the largest gains likely due to the overfitting on seen task variations. The *Baseline + Memory (specs-trained) + FHIR specs* configuration achieves the strongest gains, outperforming the baseline by 22.5% in Experiment 1 and 27.9% in Experiment 2. In Experiment 3, prompt paraphrasing yields baseline performance similar to Experiment 2, indicating prompt paraphrasing has a little impact on overall difficulty. The largest gain over baseline (18.8%) is achieved by the *Baseline + Memory (specs-trained)* configuration, though the improvement is smaller than in Experiment 2. In Experiment 4, while all memory-augmented agents outperform the baseline, the gain is smaller than previous experiments. *Baseline + Memory (no specs training)* attains the highest overall success rate (60.6%), indicating that FHIR operation-level lessons generalize across tasks but not as good as in the same task category. These trends are consistent under the light validator (Figure 5b).

### Performance consistency at different hierarchical levels

**Figure 6:**
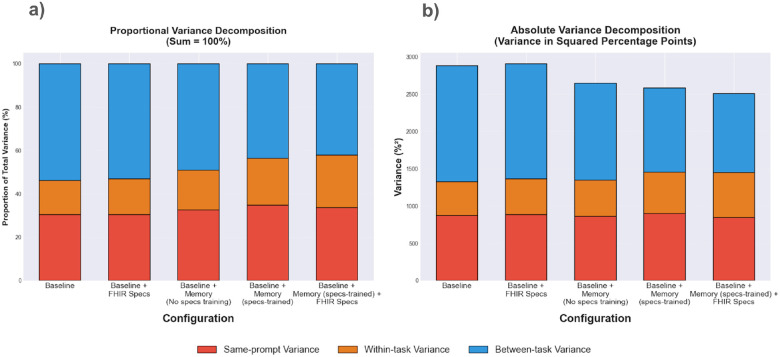
Hierarchical variance decomposition of task success across agent configurations. Figure 6ashows the proportional contribution of three variance components to total variance in task success rates for each of our five agent configurations, normalized to 100%. Figure 6b shows the absolute variance values in squared percentage points (%^2^). Same-prompt variance captures run-to-run variability for identical task descriptions, within-task variance reflects sensitivity to different task variations, and between-task variance represents performance differences across distinct tasks. Data is aggregated across all four experiments.

Given the non-deterministic nature of LLMs, LLM agents’ outputs and actions can differ even when prompted with the exact task description. This variance can be decomposed into three components (Figure 6a):

- *Same-prompt variance (run-to-run variability)*: This component captures variability across three runs of the exact same task description (prompt). Across all configurations, same-prompt variance accounts for approximately 30–35% of total variance (ranging from 845 to 899 squared percentage points) and this proportion remains stable across baseline and memory-enhanced configurations.
- *Within-task variance (prompt sensitivity)*: This component measures how performance varies across different task variations within the same task, including both task descriptions originating from the same task (e.g. different patient names or appointment dates) and semantically equivalent paraphrased prompts. Memory-enhanced configurations show a modest increase in this component, going from 15.7% of total variance for the *baseline* to ~18–24% of total variance for the memory-enhanced configurations.
- *Between-task variance (task sensitivity)*: This component reflects inherent task differences. Baseline configurations attribute over 54% of total variance to this component, compared to 42–49% for memory-enhanced configurations.

Figure 6b shows memory-enhanced configurations exhibit lower total variance overall compared to the two baseline configurations (2,503–2,643 versus 2,879–2,905 squared percentage points for baselines), with the *baseline + Memory (specs-trained) + FHIR specs* configuration having the lowest total variance at 2503 %^2^. This reduction is primarily driven by the decreased between-task variance component.

#### Error analysis

**Figure 7:**
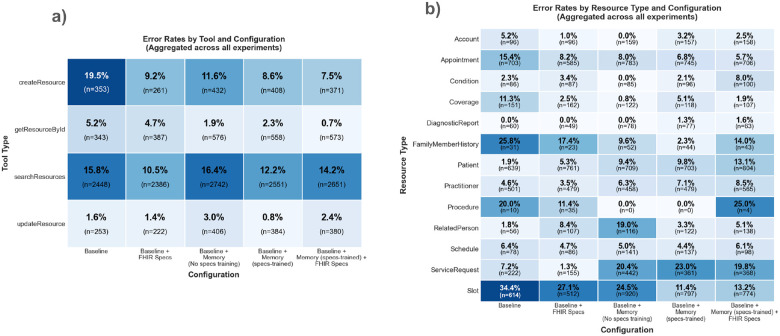
Tool and resource error rates across agent configurations. Figure 7a shows error rates for each FHIR MCP tool across the five agent configurations. Figure 7b shows error rates for tool calls targeting each FHIR resource type. Cell annotations display the error rate and total number of tool calls (n) for each tool-configuration or resource-configuration pair. Data is aggregated across all four experiments.

Figure 7a shows the percentage of erroneous FHIR MCP tool calls by MCP tools, and Figure 7b shows the percentage of erroneous tool calls by FHIR resource (e.g., errors among all tool calls involving the *Patient* resource). The tools *searchResource* and createResource tend to be more difficult and often require iterative process to get it correctly.

**Figure 8:**
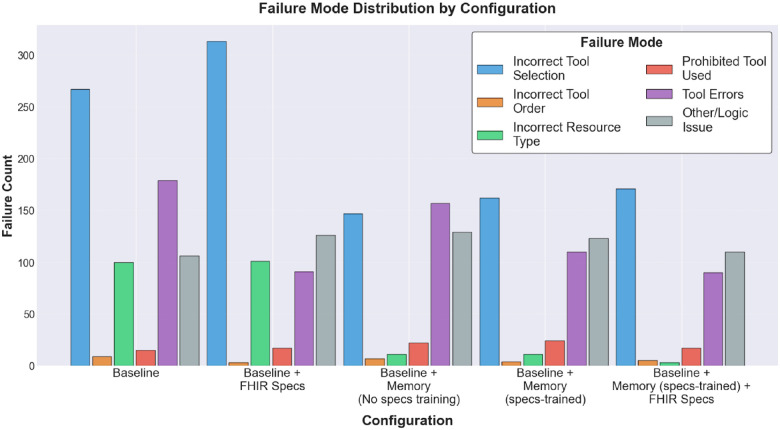
Failure mode distribution across agent configurations. Counts of six failure modes for failed task runs across the five configurations, aggregated over all four experiments. Failure modes are classified by comparing execution logs against task metadata. Individual failed task runs may exhibit multiple failure modes simultaneously.

While Figure 7 shows error rates across all agent runs in our experiments (even successful agent runs may include intermediate tool-call errors that the agent later corrects), Figure 8 analyzes failed agent runs and decomposes failures into various failure-modes derived from task metadata and execution traces, with each agent run potentially triggering multiple flags. Across all experiments, the *baseline* and *baseline + FHIR specs* configurations show the highest counts for incorrect tool selection and incorrect resource type relative to the memory-accessing configurations. Furthermore, the failure flags are defined as follows:

- *Incorrect tool selection:* the agent never executed a tool set that satisfies the task’s required tool-call templates (e.g. missing one or more required tools). For example, a task requires a *searchResources* call followed by a *updateResource* call, but the run only calls *getResourceById* and *updateResource*.
- *Incorrect tool order:* the agent executed the required tool calls, but not in the required order (no required tool call sequence appears as a subsequence of the observed tool order of the agent run). For example, a task requires the following tool call sequence [*searchResources* → *getResourceById* → *updateResource*], but the run calls *updateResource* before retrieving the target resource.
- *Incorrect resource type*: only evaluated when tool selection passes; the agent’s tool calls never touch all required FHIR resource types for the task (resource types are extracted from tool inputs/outputs). For example, a scheduling task requires interacting with the FHIR resources *Patient* and *Appointment*, but the execution logs show only tool calls to *Patient*.
- *Prohibited tool used:* the run includes a tool that the task explicitly disallows. For example, a patient record update task forbids *deleteResource*, but the agent calls it anyway.
- *Tool errors:* at least one tool call returns an error. For example, a *createResource* call fails with a validation error due to missing required fields.
- *Other/logic issues:* serves as a catch-all bucket for failed runs where none of the above failure flags fire. In other words, the agent run failure is not explained by our metadata-based checks.

#### Number of tool calls per configuration

We compare FHIR MCP tool call distributions per configuration across 4 outcome categories:

- *Overall Success*: the total number of tool calls per task among successfully completed tasks.
- *Overall Failure,* the total number of tool calls per task for failed tasks.
- *Error Success:* the number of erroneous tool calls per task for successfully completed tasks.
- *Error Failure*: the number of erroneous tool calls per task for failed tasks.

**Figure 9:**
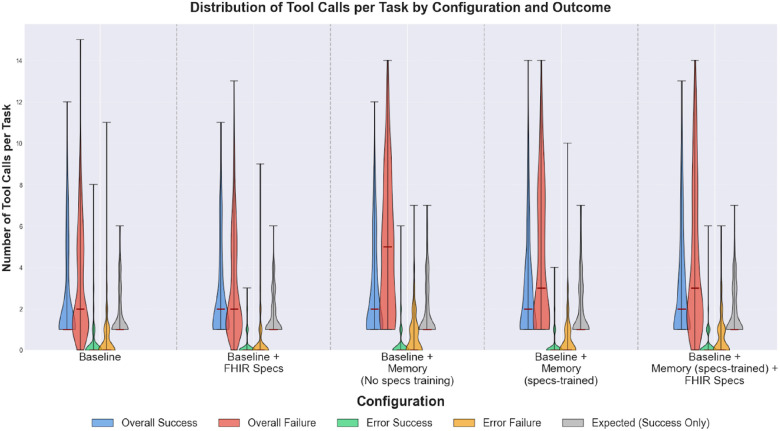
Distribution of tool calls per task by configuration and outcome. Violin plots show the distribution of FHIR MCP tool calls per task across the five agent configurations, grouped by task outcome. For each configuration, five distributions are shown: Overall Success (total tool calls in successful runs), Overall Failure (total tool calls in failed runs), Error Success (erroneous tool calls in successful runs), Error Failure (erroneous tool calls in failed runs), and Expected (minimal required tool calls per task, restricted to tasks completed successfully by each configuration, shown as reference). Violin width indicates frequency density, with median values marked by red horizontal lines. Data aggregated across all four experimental settings.

We also include, as a reference, the distribution of the expected minimal number of tool calls per task, restricted to the task variations successfully completed by each configuration. These expected values are task-specific and treated as part of the task metadata. Figure 9 shows successful task runs have similar overall tool-call distributions. In contrast, failed task runs show the highest observed disparities in overall tool calls distributions across configurations. The two baseline configurations show median overall tool calls of 2 (IQRs of 4) for failed tasks, while memory-enhanced configurations show higher medians.

#### Token usage Analysis

**Figure 10:**
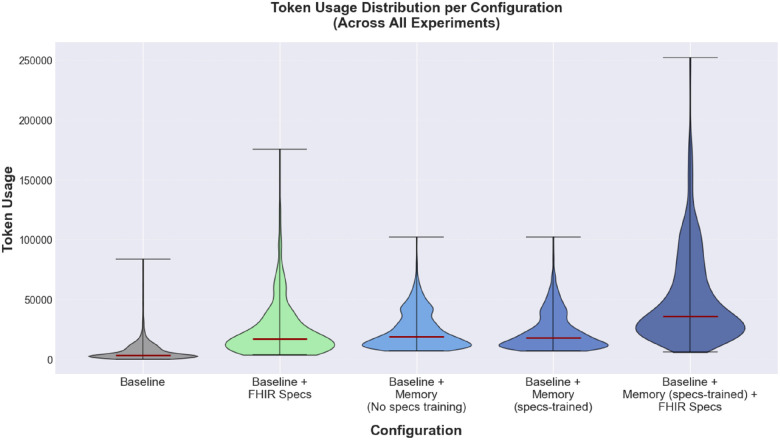
Token usage distribution across agent configurations. Violin plots show the distribution of token usage per task run across configurations, aggregated over the four experimental settings. Medians are marked in red. Violin width indicates frequency density, and extrema show the full range of observed values.

Figure 10 shows the overall token-usage distributions across all task-description runs. The memory-based configurations consistently show higher median token usage than the bare baseline, as expected. The *baseline + FHIR specs* configuration shows a wider spread in token usage than the baseline and the two trained-memory configurations. The *baseline + Memory (specs-trained) + FHIR specs* configuration has the highest median token usage and the widest spread among all configurations.

**Figure 11:**
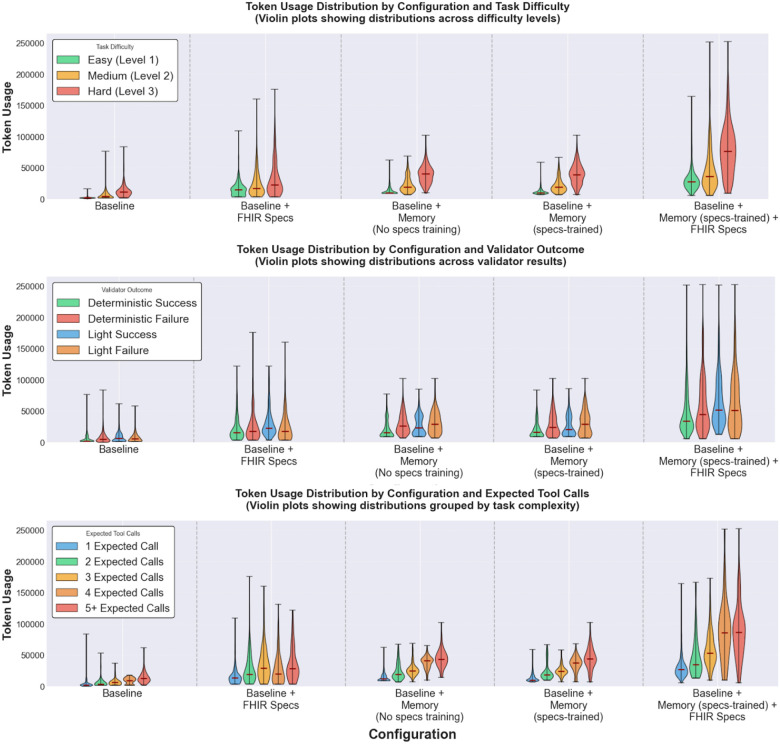
Token usage distribution by configuration and task characteristics. Figure 11a shows token usage distributions grouped by task difficulty levels (1, 2, or 3). Figure 11bshows distributions grouped by validation outcome (deterministic success/failure and light validation success/failure). Figure 11c shows distributions grouped by expected minimal tool calls (1, 2, 3, 4, or 5+ calls). For each subplot, violin plots display token usage per task run across the five configurations, with medians marked in red. Violin width indicates frequency density, and extrema show the full range. Data is aggregated across all four experimental settings.

Figure 11 shows token usage by difficulty, expected tool calls, and expected outcomes. Token usage increases with task difficulty across configurations, with median token usage rising from difficulty 1 to 3. We observe a similar pattern when grouping by the expected minimal number of tool calls per task. Median token usage scales with expected tool-call requirements across configurations, with *baseline + FHIR Specs* being the sole exception. Finally, across all configurations, failed runs have higher median token usage than successful runs.

**Figure 12:**
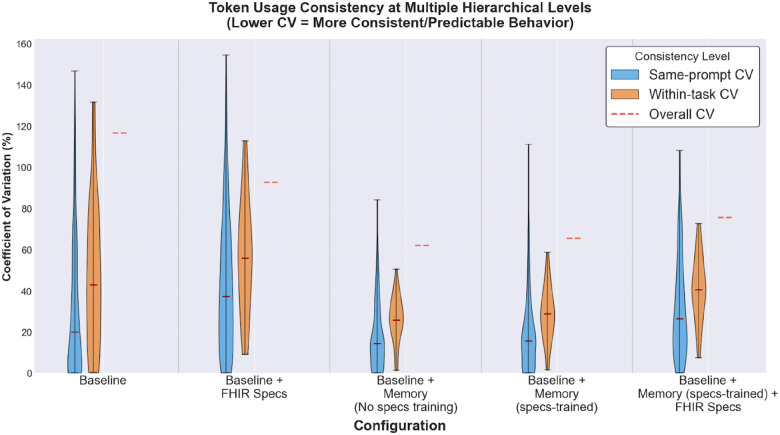
Token usage consistency across hierarchical levels. Coefficient of variation (CV) of token usage for each configuration at three hierarchical levels. Same-prompt CV (blue violins) captures consistency across reruns of identical task descriptions. Within-task CV (orange violins) reflects consistency across different variations of the same task. Overall CV (red dashed lines) represents aggregate consistency across all tasks. Lower CV indicates more consistent and predictable token usage. Data is aggregated across all four experimental settings.

Figure 12 quantifies token-usage consistency by computing the coefficient of variation (CV) (standard deviation of token usage values divided by the mean) at multiple hierarchical levels, where lower CV indicates more stable token usage. The two memory-enhanced configurations, *baseline + Memory (no-specs-training)* and *baseline + Memory (specs-trained)*, show the lowest median CVs across all three levels, with both below the baseline variants. In contrast, the two baseline configurations have the highest median CVs and the widest spreads. The *baseline + Memory (specs-trained) + FHIR specs* configuration shows a more mixed pattern. Its within-task and overall CV values are higher than the two trained-memory configurations, but overall, it remains more stable than the bare baseline. However, this comes with a clear cost in token usage, which is substantially higher for this configuration.

## Discussion

Our study makes three key contributions. First, we introduce an extensible sandbox for evaluating LLM agents on realistic FHIR-based workflows. Built around a resettable HAPI FHIR server, generic CRUD tools, and deterministic state-based validation, the sandbox captures core challenges of real-world clinical interoperability that have been underexplored in prior work. Second, beyond the sandbox as a system artifact, we present a reproducible and reusable evaluation methodology for constructing reliable and extensible benchmarks for agent evaluation. This framework provides general principles for task design, execution control, and outcome validation, enabling future benchmarks to be developed consistently across different workflows, models, and settings. Third, through systematic experiments conducted within this sandbox, we offer empirical evidence on the role of memory in agent performance. Our results show that while current LLM agents remain unreliable for end-to-end FHIR task execution, memory-enhanced approaches can meaningfully improve both task success rates and operational reliability.

Earlier work such as FHIR-AgentBench^22^ demonstrates that even strong ReAct-style agents achieve only modest correctness in read-only question answering, our results show that similar limitations persist and are amplified when agents must create or modify FHIR resources, which involves common real-world use cases such as appointment scheduling, order management, and clinical documentation updates. In particular, we observe resource-type confusion and incorrect tool sequencing as dominant failure modes across both retrieval and write-oriented tasks, highlighting a fundamental challenge in translating natural language requests into structured, schema-constrained clinical actions. Additionally, MedAgentBench reports strong performance (85.33%) on query-style tasks but a marked degradation on action tasks (54.00%)^10^, highlighting the difficulty of correct FHIR resource manipulation. Similarly, we observe frequent instruction-following and tool-use failures, but our analysis further reveals strategic errors, such as incorrect tool selection and resource-type confusion, as dominant failure modes. Unlike MedAgentBench, which relies solely on human-curated references for evaluation, we found it challenging to define accurate gold standards for FHIR action tasks due to the high flexibility of the FHIR standard. Variability across FHIR implementation guides poses a practical challenge for evaluation prior to deployment, as an agent may generate resources that are valid under core FHIR R4 yet fail to satisfy stricter, Implementation Guides (IG)-specific constraints. While our use of soft validators helps surface such cases, these issues reflect real-world interoperability complexity rather than benchmark artifacts, underscoring the difficulty of deploying agents across heterogeneous FHIR profiles. As LLMs continue to improve, human–machine collaborative approaches should be considered in benchmark design.

Execution log analysis reveals recurring failure patterns that underscore the difficulty of reasoning directly over FHIR. Agents often generate resources that are syntactically valid but semantically incorrect, miscompute temporal constraints such as relative dates or time windows, or retrieve incorrect entities due to overly broad or underspecified queries and limited recovery when initial searches fail. Additional errors arise from the use of outdated or non–FHIR R4 conventions, including deprecated fields and reference patterns. Failure-mode analysis further shows that the primary benefit of long-term memory lies in reducing strategic execution errors—such as incorrect tool or resource-type selection—rather than isolated low-level tool mistakes. Memory-enabled agents exhibit better alignment between task requirements and action sequences, accounting for much of their performance advantage over baseline agents. In contrast, runtime access to specifications alone mitigates some low-level errors but fails to address these higher-level failure modes, explaining its limited impact on end-to-end task success.

Our experiments show Reflexion-based memory consistently improves task success, particularly for workflows that recur with variation. This is an important property for clinical operations, which often follow standard patterns but differ in patient-specific details (i.e. same task but multiple task variations). These findings align with prior work on reflection-based agents including Reflexion^12^, and ReflecTool^23^. For example, ClinicalAgent Bench (CAB) is a benchmark of 18 tasks grouped into five capability dimensions, designed to evaluate agents to solve tasks by selecting and using tools from a broad clinical toolbox^23^. Their method builds long-term memory by storing successful tool-use trajectories and distilling tool-specific lessons generated during optimization^23^ (analogous to our training phase). On CAB, with their Qwen2-72B^24^ setting, ReflecTool (candidate-selection) improves the overall average score from 53.31 (ReAct) to 59.66 (ReflecTool), and in a tool-selection error analysis, task-level tool-selection errors drop from 4.03 (ReAct) to 0.08 (ReflecTool with candidate-selection)^23^.In addition, memory augmentation improves agent reliability, yielding more consistent task performance, more stable token usage, and lower tool error rates. While memory does not reduce run-to-run stochasticity, it significantly lowers between-task variance, resulting in more predictable behavior across tasks of varying difficulty. Such consistency is critical in healthcare settings, where unpredictable failures can undermine trust and safety. However, generalization to unseen tasks is still limited, with performance gains shrinking on held-out tasks, indicating that broader and more diverse training coverage is required before deployment in open-ended clinical settings.

Runtime access to FHIR specifications alone does not meaningfully improve end-to-end task success, despite modest reductions in tool error rates. In contrast, agents whose long-term memory was trained with specification-grounded reflections achieve both lower tool error rates across all core FHIR tools and higher overall task success. In addition, specs-trained memory provides higher-level strategic guidance beyond tool-specific rules, helping agents not only avoid low-level errors but also execute workflows more successfully. This suggests that, for real-world deployment, it might be more effective to distill clinical standards and schema constraints into task-level heuristics during Reflexion-style training rather than relying on agents to consult raw documentation during live workflows.

## Conclusion

In this work, we developed a modular sandbox for benchmarking LLM agents on realistic FHIR-based clinical workflows and demonstrated a systematic framework for evaluating action-oriented clinical tasks. By integrating a resettable FHIR environment with deterministic validation, our approach enables rigorous assessment of agent behavior beyond read-only question answering.

Using this framework, we show that current LLM agents remain unreliable for end-to-end execution of structured clinical workflows, particularly in multi-step tasks requiring persistent state changes. Long-term, reflexion-based memory substantially improves task success and reduces strategic failures such as incorrect tool selection and resource-type confusion, whereas runtime access to FHIR specifications alone provides limited benefit.

These findings highlight both the promise and limitations of memory-augmented agent design in clinical settings and underscore the importance of action-oriented evaluation before deployment in real-world healthcare systems. The proposed sandbox provides a foundation for more systematic assessment of agent reliability and offers a pathway toward safer and more predictable AI-assisted clinical workflows.

## Supplementary Files

This is a list of supplementary files associated with this preprint. Click to download.

- FHIRAgentEvalSupplementaryFiles.pdf

## Figures and Tables

**Table 1 |:** Design choices for the five agent configurations used in the four experimental settings.

Configuration	FHIR CRUD (MCP)	FHIR Specs at runtime	Memory at runtime (trained with no specs)	Memory at runtime (trained w/ specs)
*Baseline*	✓			
*Baseline + FHIR spec*	✓	✓		
*Baseline + Memory (no specs training*	✓		✓	
*Baseline + Memory (specs-trained)*	✓			✓
*Baseline + Memory (specs-trained) + FHIR specs*	✓	✓		✓

## Data Availability

The benchmark and sandbox environment are available at: https://github.com/YoussefMkst/FHIR-AgentEval/tree/main/environment/data

## Code availability

The experiment code for the Reflexion-inspired training, evaluation sandbox setup, modular benchmark tasks, and evaluation of different agent configurations is available at: https://github.com/YoussefMkst/FHIR-AgentEval
